# Inhibition of intestinal polyp growth by oral ingestion of bovine lactoferrin and immune cells in the large intestine

**DOI:** 10.1007/s10534-014-9747-2

**Published:** 2014-05-28

**Authors:** Masaaki Iigo, David B. Alexander, Jiegou Xu, Mitsuru Futakuchi, Masumi Suzui, Takahiro Kozu, Takayuki Akasu, Daizo Saito, Tadao Kakizoe, Koji Yamauchi, Fumiaki Abe, Mitsunori Takase, Kazunori Sekine, Hiroyuki Tsuda

**Affiliations:** 1 Nanotoxicology Project, Nagoya City University, Nagoya, Japan; 2 Department of Molecular Toxicology, Nagoya City University Graduate School of Medical Sciences, Nagoya, Japan; 3 National Cancer Center Central Hospital (as of 2005), Tokyo, Japan; 4 Morinaga Milk Industry Co., Ltd., Minatoku, Japan

**Keywords:** Ingestion of bovine lactoferrin, Immune function, Human intestine, Ancillary study of a human clinical trial

## Abstract

Studies using animal models have demonstrated that ingestion of bovine lactoferrin (bLF) inhibits carcinogenesis in the colon and other organs of experimental animals. As a result of these studies, a blinded, randomized, controlled clinical trial was conducted in the National Cancer Center Hospital, Tokyo, Japan to determine whether ingestion of bLF had an effect on the growth of colorectal polyps in humans. Patients with colorectal polyps ≤5 mm diameter and likely to be adenomas ingested 0, 1.5, or 3.0 g bLF daily for 1 year. Ingestion of 3.0 g bLF suppressed the growth of colorectal polyps and increased the level of serum human lactoferrin in trial participants 63 years old or younger. The purpose of the present study was to investigate correlations between immune parameters and changes in polyp size. Trial participants with regressing polyps had increased NK cell activity, increased serum hLF levels (indicating increased neutrophil activity), and increased numbers of CD4+ cells in the polyps. These findings are consistent with a correlation between higher immune activity and suppression of colorectal polyps. In addition, participants with regressing polyps had lower numbers of PMNs and increased numbers of S100A8+ cells in the polyps, consistent with a correlation between lower inflammatory potential in the colon and suppression of colorectal polyps. Trial participants ingesting bLF had increased serum hLF levels, a possible increase in systemic NK cell activity, and increased numbers of CD4+ and CD161+ cells in the polyps. Taken together, our findings suggest that bLF suppressed colorectal polyps by enhancing immune responsiveness.

## Introduction

Lactoferrin (LF) is an approximately 80 kDa protein present at high levels in milk, tear film, and neutrophil granules and at moderate to high levels in upper airway and seminal fluids (reviewed in Alexander et al. 2012). Endogenous LF is an important component of the innate immune system and its primary role is the non-lethal, non-inflammatory removal of microbial pathogens from cells and tissues.

The activity of LF ingested by adults is distinct from the activity of endogenous LF (see Alexander et al. 2012): Ingestion of bovine lactoferrin (bLF) has been shown to induce the expression of cytokines in the intestine, enhance the activity of immune effector cells, and inhibit carcinogenesis in the colon and other organs of experimental animals (reviewed in Tsuda et al. 2010).

As a result of the studies demonstrating an inhibitory effect of ingested bLF on colon carcinogenesis, a randomized, controlled clinical trial beginning in 2002 and ending in 2006 was conducted in the National Cancer Center Hospital, Tokyo, Japan to determine whether ingestion of bLF had an effect on the growth of adenomatous colorectal polyps in humans (Kozu et al. 2009). Briefly, trial participants ingested 0, 1.5, or 3.0 g bLF daily for 1 year: The size of colorectal polyps was measured prior to the beginning and at the end of the trial; peripheral blood samples were collected at the commencement of the trial and at the end of 3, 6, 9, and 12 months (the end of the trial), and T cell subpopulation numbers, natural killer cell activity and number, neutrophil number, and the serum levels of interleukin-18, interferon-gamma, and human lactoferrin (hLF) were measured; normal mucosa, close to the polyp, was collected prior to the beginning and at the end of the trial and polyps were collected at the end of the trial for RNA extraction and histological examination. The trial reported that ingestion of 1.5 g bLF had no significant effect on any of the parameters measured. Ingestion of 3.0 g bLF, however, had two significant effects: (i) the growth of colorectal polyps was inhibited in trial participants 63 years old or younger and (ii) the level of hLF in the serum was increased in trial participants 63 years old or younger (Kozu et al. 2009). The authors of the Tokyo-trial concluded that ingestion of 3.0 g bLF daily for one year inhibited the growth of adenomatous colorectal polyps and probably acted via modulation of immune system function.

Studies with animal models that have shown ingestion of bLF promoted immune system activity agree with the conclusions drawn from the Tokyo-trial: bLF-mediated inhibition of colorectal polyp growth probably acted via modulation of immune system function. The present study was undertaken to determine if immune cell parameters in the Tokyo-trial participants could be correlated with the changes in colorectal polyp size observed in the trial.

## Methods

### The Tokyo trial

A blinded, randomized, controlled clinical trial beginning in 2002 and ending in 2006 was conducted in the National Cancer Center Hospital, Tokyo, Japan to determine if ingestion of bLF would inhibit the growth of precancerous, adenomatous colorectal polyps in human patients. A complete description of the Tokyo-trail design, participants, interventions, outcomes, sample size, randomization, blinding, statistical methods, characteristics of the intent-to-treat population at the commencement of the trial, and the CONSORT flowchart are presented in Kozu et al. (2009).

### Ethics statement

The Tokyo-trial was initiated after approval by the Ethical Committee of the National Cancer Center Hospital, Tokyo, Japan and is registered in the University Hospital Medical Information Network Clinical Trials Registry (UMIN-CTR; http://www.umin.ac.jp/ctr/index.htm) Tokyo, Japan, number C000000182. All trial participants provided written informed consent.

### NK activity in the blood

Peripheral blood was collected from participants at the beginning of the trial and at 3, 6, 9, and 12 months (at the end of the trial). Blood samples used to measure NK cell activity were diluted with saline, and lymphocytes were separated from the diluted blood samples using Ficoll-Conray solution (relative density, 1.077; IBL). The entire lymphocyte population (containing effector cells) was washed twice with PBS. Cell killing activity was then measured using K-562 cells labeled with ^51^Cr as target cells. Target cells (1 × 10^6^/mL, 10 µL) and effector cells (1 × 106/mL, 200 µL) were mixed and incubated at 37 °C, 5 % CO_2_ for 3.5 h. The medium was then removed and clarified by centrifugation, and soluble ^51^Cr released by killed K- 562 cells was measured with a gamma-counter (1470 Wizard, Perkin- Elmer Life and Analytical Sciences).

### ELISA for serum lactoferrin

Peripheral blood was collected from trial participants as noted above. Microtiter plates were coated with a mouse anti-hLF antibody (mouse, clone: 2B8, IgG1, Advanced Immuno Chemical) and incubated at 5 °C overnight. To block the wells, 250 µL of 0.5 % gelatin in PBS was added to the wells and the plate was incubated at 37 °C for 1 h. 100 µL of sample was applied to the blocked wells and the plate was incubated at 5 °C overnight. Captured hLF was detected using horseradish peroxidase–labeled polyclonal antibodies against hLF (rabbit; Cappel) and visualization was done using o-phenylenediamine (Sigma). The minimal detectable concentration of hLF was 200 pg/mL; the minimal detectable concentration of bLF (using the hLF ELISA) was >20 µg/mL. The ELISA for bLF, using a specific antibody against bLF developed by Morinaga Milk Industry, was done similarly. Briefly, microtiter plates were coated with capture antibody (anti-bLF rabbit polyclonal, Morinaga Milk Industry) and blocked, and 100 µL sample was applied. Captured bLF was detected using a biotin-labeled anti-bLF polyclonal antibody (rabbit; Nacalai, Japan) and visualized with horseradish peroxidase–labeled streptavidin (Zymed) and o-phenylenediamine. The minimal detectable concentration of bLF was 500 pg/mL; the minimal detectable concentration of hLF (using the bLF ELISA) was >20 µg/mL.

### Collection of tissue samples

Target polyps and tissue sample sites were located at the most proximal sites of the right colon (cecum to transverse colon) and the left colon (descending colon to rectum). Prior to the start of the trial a small tissue sample was collected from normal mucosa close to the target polyp. At the end of the trial period, a final colonoscopic examination was performed and a second normal tissue sample was collected and target polyps (and all other premalignant and malignant growths found) removed. Afer collection, tissue and polyp samples were immediately transferred to the laboratory on ice and divided into halves; RNA was extracted from one half, and the other half was immediately fixed in buffered formalin, embedded in paraffin, and processed for histopathological examination.

### Counting polymorphonuclear leukocytes in the target polyps

Sections stained with H&E were used to count PMNs. PMNs were counted in the stroma of adenomatous polyps. Only polyp sections containing at least 5 atypical adenoma glands and mucosa propria to a depth equal to at least the average gland diameter (d) were used. PMNs in the stroma of 5 glands and the underlying mucosa propria to depth “d” were counted. The area was measured using an image analysis system, Image Processor for Analytical Pathology (IPAP, Sumika Technos Corp., Osaka, Japan).

### Immunohistochemistry

CD4+, CD161+, CD66b+, S100A8+, and S100A9+ cells were detected using anti-CD4 polyclonal antibody (A429, Rabbit polyclonal) diluted 1:125, anti-CD161 monoclonal antibody (abcam, Mouse monoclonal IgG2b) diluted 1:100, ant-CD66b monoclonal antibody (Bioleggend, Mouse IgM) diluted 1:125, anti-S100A8 monoclonal antibody (MAC 387, Gene Tex, Mouse monoclonal IgG1) diluted 1:500, and anti-S100A9 monoclonal antibody (EPR3555, Gene Tex, Rabbit monoclonal IgG) diluted 1:5000. Positive cells were visualized with secondary antibody and Vectastain elite ABC kit. Slides were prepared according to Leica microsystems protocol: DeWax 5 min, high temperature antigen unmasking, 5 % H_2_O_2_ 5 min, blocking 5 min, first antibody 60 min, second antibody 30 min, DAB 30 min, hematoxylin 5 min.

### Statistics

The data for NK activity in the blood and serum hLF levels was analyzed using Dunnett’s test. The data for PMN cells in the polyps was analyzed using Dunnett’s test. The data for markers expressed primarily by a single cell type (CD66b, CD4 and CD161) was analyzed using Dunnett’s test. The data for markers expressed primarily by multiple cell types (S100A8+ and S100A9+) was analyzed using Hsu’s MCB test. Pearson coefficients of correlation were calculated to determine the degree of association between polyp size and immune cells in the polyp. Levels of significance were set at 0.05 (two-sided) for all statistical analyses.

## Results and discussion

### Correlation of immune parameters with polyp growth

#### Systemic NK cell activity and blood PMN counts and serum hLF

**Fig. 1:**
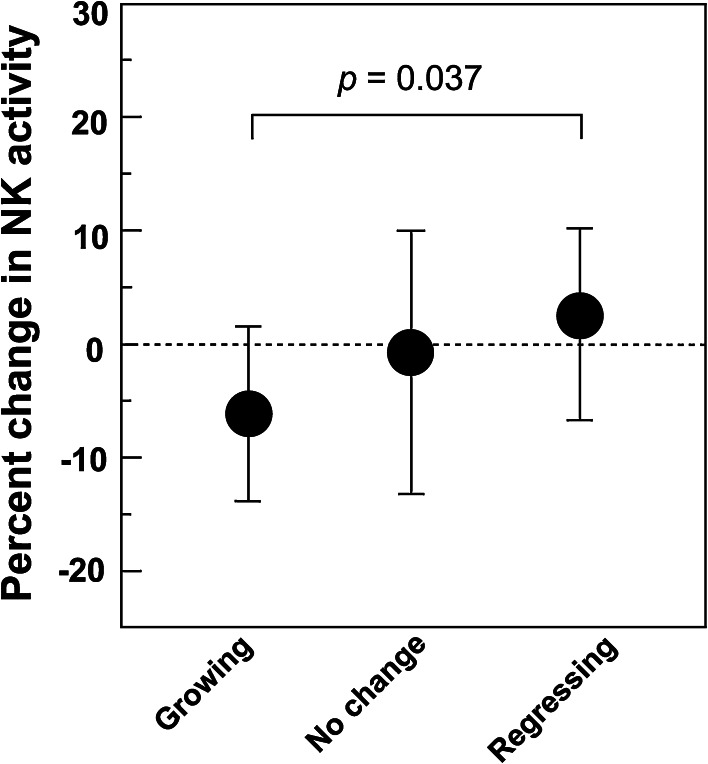
Systemic NK cell activity and polyp growth. Polyp size and NK activity in peripheral blood samples were measured prior to the beginning and at the end of the trial. 19 participants had target polyps that had increased in size by 20 % or more (growing polyps), 11 participants had target polyps that had decreased in size by 20 % or more (regressing polyps), and the remaining 73 participants had polyps that were diagnosed as having no change in size. The relative change in NK activity in the blood of the trial participants at the end of the one-year trial period was significantly higher in participants with regressing polyps compared to participants with growing polyps. This figure is adapted from Fig. S2, Kozu et al. (2009), Effect of Orally Administered Bovine Lactoferrin on the Growth of Adenomatous Colorectal Polyps in a Randomized, Placebo-Controlled Clinical Trial

**Fig. 2:**
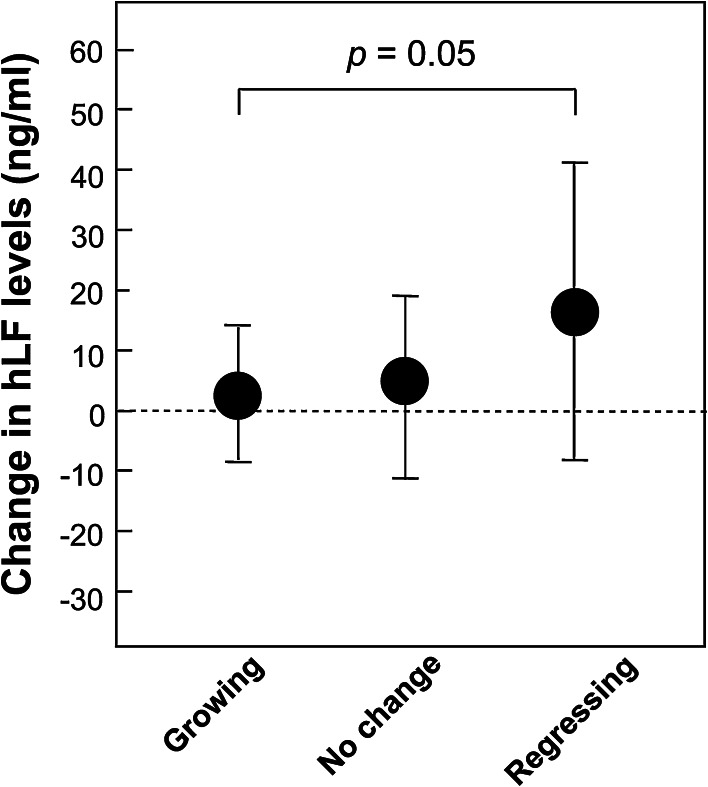
Serum hLF levels and polyp growth. Polyp size and hLF levels in peripheral blood samples were measured prior to the beginning and at the end of the trial. 19 participants had target polyps that had increased in size by 20 % or more (growing polyps), 11 participants had target polyps that had decreased in size by 20 % or more (regressing polyps), and the remaining 73 participants had polyps that were diagnosed as having no change in size. The change in hLF levels in the blood of the trial participants at the end of the one-year trial period was significantly higher in participants with regressing polyps compared to participants with growing polyps

As previously reported by Kozu et al. (2009), for the Tokyo-trial participants there was a statistically significant correlation between polyp growth during the one-year trial period and systemic NK activity and between polyp growth and levels of serum hLF. Polyp size, NK activity, and hLF levels in peripheral blood samples were measured prior to the beginning and at the end of the trial. 19 participants had target polyps that had increased in size by 20 % or more (growing polyps), 11 participants had target polyps that had decreased in size by 20 % or more (regressing polyps), and the remaining 73 participants had polyps that were diagnosed as having no change in size. The relative change in NK activity in the blood of the trial participants at the end of the one-year trial period was significantly higher in participants with regressing polyps compared to participants with growing polyps (Fig. 1). The change in hLF levels in the blood of the trial participants at the end of the one-year trial period was also significantly higher in participants with regressing polyps compared to participants with growing polyps (Fig. 2). Importantly, the overall number of neutrophils in the serum was not increased in patients with regressing polyps (data not shown). Therefore, since serum hLF is derived primarily from neutrophils (Ambruso et al. 1984; Gessler et al. 2004; Lash et al. 1983; van der Strate et al. 1999; Brown et al. 1986, 1983), the increase in serum hLF levels suggest that in patients with regressing polyps, serum neutrophils were more active than serum neutrophils in participants with growing polys. Taken together, these data suggest that, on average, trial participants with regressing polyps had higher levels of immune system activity, as manifested by higher levels of systemic NK cell and neutrophil activity, than participants with growing polyps.

#### Polyp-associated CD4+ cells

**Fig. 3:**
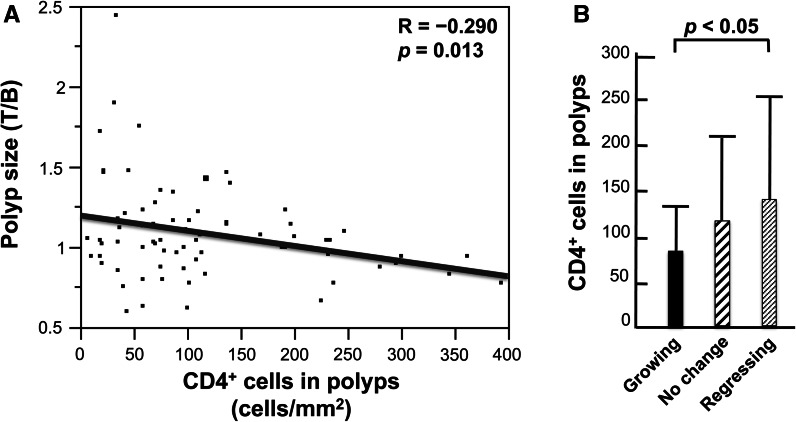
Polyp-associated CD4+ cells and polyp growth. At the end of the trial, a final colonoscopic examination was performed. All target polyps were then removed and processed for histological examination. 91 polyps were histologically diagnosed, and the density of CD4+ cells could be determined by immunohistochemistry in 72 of these polyps: 28 target polyps had increased in size by 10 % or more (growing polyps), 16 target polyps had decreased in size by 10 % or more (regressing polyps), and the remaining 28 polyps were diagnosed as having no change in size. **a** There was a significant inverse correlation between the numbers of CD4+ cells in the polyps and the change in polyp size during the one-year trial period. **b** There was a significantly higher density of CD4+ cells in regressing polyps compared to growing polyps

There was a significant inverse correlation between the change in polyp size and the density of CD4+ cells in the polyp (Fig. 3a), and regressing polyps contained a significantly higher density of CD4+ cells than growing polyps (Fig. 3b). This is consistent with the key role helper T cells play in the immune system and suggests that activation of immune cells by helper T cells may play a role in inhibiting the growth of colorectal polyps. The increase of CD4+ cells in regressing polyps is consistent with the proposition stated above that, on average, participants with regressing polyps had higher levels of immune system activity than participants with growing polyps.

#### Polyp-associated CD161+ cells

CD161+ cells (primarily NK cells) were not increased in regressing polyps (data not shown). This result would appear to contravene the proposition that colorectal polyps were suppressed in trial participants with higher immune system activity. However, the most likely reason that NK cells were not increased in regressing polyps is that in general pre-cancerous polyp cells are essentially normal and are not targeted by the immune system. Therefore, immune cells involved in immunosurveillance and removal of transformed cells, such as NK cells, will be active in the polyps only sporadically, as pre-cancerous cells become transformed from pre-cancerous cells into cells with increased tumorigenic potential and become targets of the immune system. Consequently, even in patients with higher systemic NK cell activity, a sustained increase of NK cells in pre-cancerous colorectal polyps would not be expected to occur.

#### Polyp-associated PMN leukocytes

**Fig. 4:**
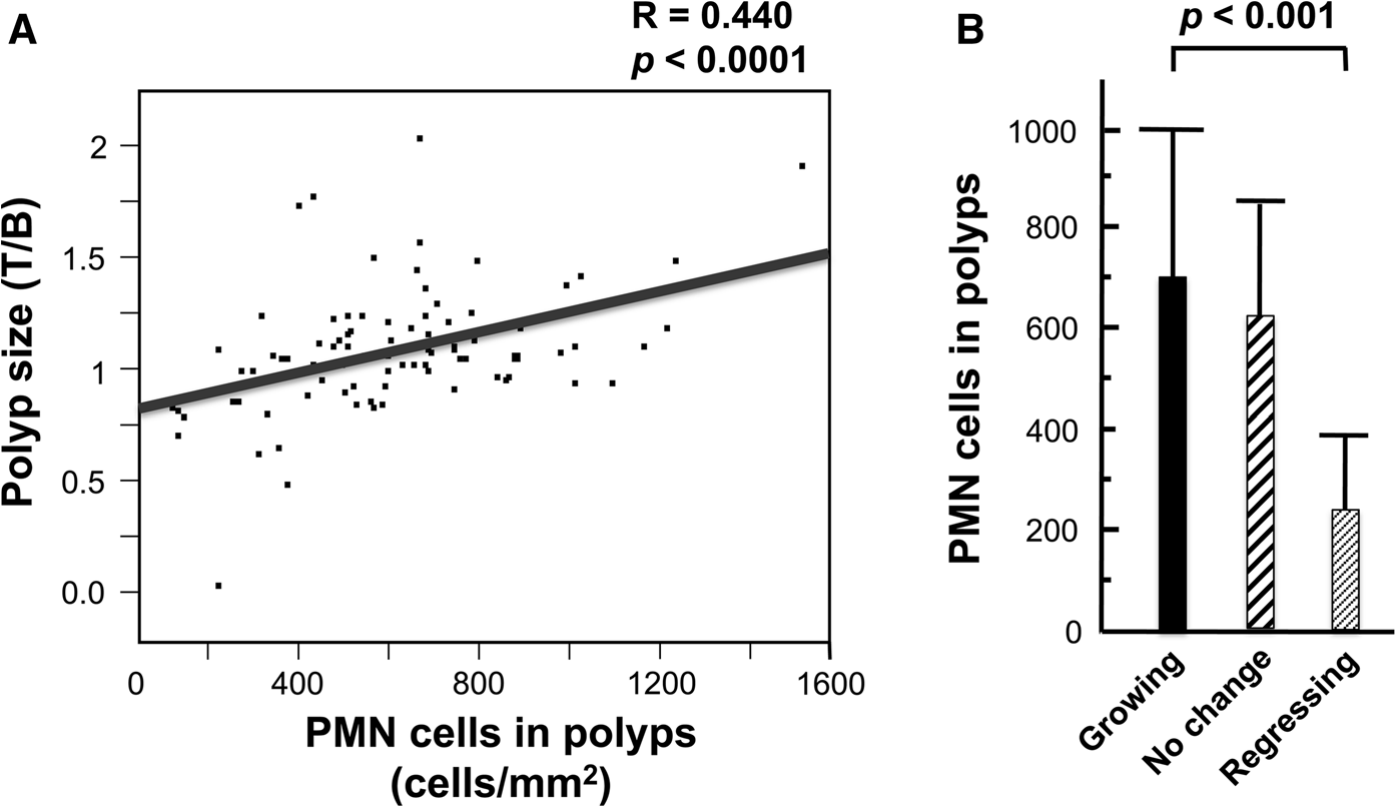
Polyp-associated PMN leukocytes and polyp growth. At the end of the trial, a final colonoscopic examination was performed. All target polyps were then removed and processed for histological examination. 91 polyps were histologically diagnosed, and the density of PMNs in H&E stained sections could be determined in 88 of these polyps: 19 participants had target polyps that had increased in size by 20 % or more (growing polyps), 11 participants had target polyps that had decreased in size by 20 % or more (regressing polyps), and the remaining 58 participants had polyps that were diagnosed as having no change in size. **a** There was a significant correlation between polyp size and the numbers of PMN cells in the polyps. **b** There was a significantly higher density of PMN cells in growing polyps compared to regressing polyps. *Panel A* is adapted from Fig. 3, Kozu et al. (2009), Effect of Orally Administered Bovine Lactoferrin on the Growth of Adenomatous Colorectal Polyps in a Randomized, Placebo-Controlled Clinical Trial

As previously reported by Kozu et al. (2009), there was a significant correlation between polyp size and the density of PMNs in the polyp (Fig. 4a), and growing polyps contained a significantly higher density of PMNs than regressing polyps (Fig. 4b). While this result would also appear to contravene the proposition that colorectal polyps were suppressed, as manifested by their size and growth, in trial participants with higher immune system activity, three considerations demonstrate that the decrease of PMNs in regressing polyps is not at variance with this proposal. First, elevated immune system activity would encompass both immune cells that target transformed cells, such as NK cells, macrophages, and cytotoxic T cells, as well as immune cells that do not specifically target transformed cells such as PMNs. Therefore, elevated immune activity would not be expected to result in an increase of PMNs in regressing polyps. Second, as noted above, pre-cancerous polyp cells transform into cells with increased tumorigenic potential and become targets of the immune system only sporadically; therefore, signals that would induce PMN infiltration into the polyp (Bhatnagar et al. 2010) would be infrequent and brief. Third, infiltration of PMNs into a tumor site can enhance tumor growth (van den Tol et al. 2007; Wislez et al. 2007; Wada et al. 2007; Queen et al. 2005); therefore, decreased infiltration PMNs into colon polyps may partially account for the correlation between decreased polyp size and decreased PMN density in the polyp.

#### Polyp-associated CD66b+ cells

There was no correlation between polyp growth and the density of CD66b+ cells in the polyp (data not shown). CD66b is primarily expressed by activated granulocytes. This result indicates that activated granulocytes are not increased in regressing polyps and is consistent with the results obtained for activated NK cells: Signals that would activate granulocytes in the polyps, such as activated NK-mediated lysis of transformed cells (Bhatnagar et al. 2010), would be present only transiently in the polyps, and therefore, a sustained increase of activated granulocytes would not be expected to occur in these polyps.

#### Polyp-associated S100A8+ and S100A9+ cells

**Fig. 5:**
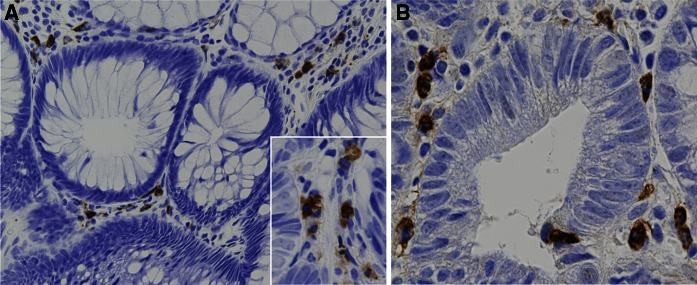
S100A8+ and S100A9+ cells. Typical S100A8+ (**a**) and S100A9+ (**b**) cells in colorectal polyps are shown

**Fig. 6:**
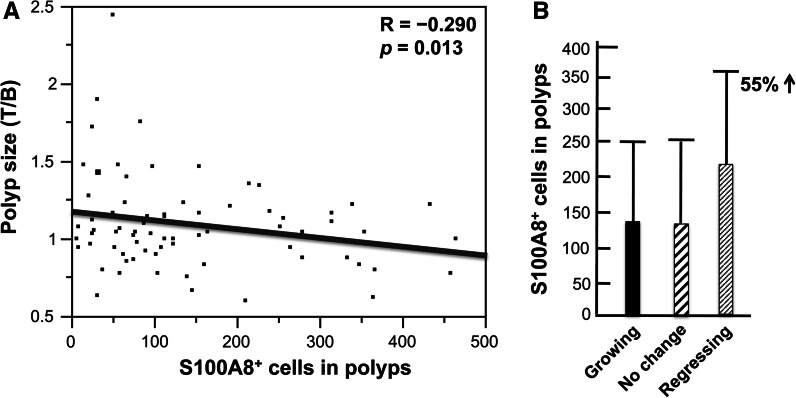
Polyp-associated S100A8+ cells and polyp growth. At the end of the trial, a final colonoscopic examination was performed. All target polyps were then removed and processed for histological examination. 91 polyps were histologically diagnosed, and the density of S100A8+ cells could be determined by immunohistochemistry in 73 of these polyps: 29 participants had target polyps that had increased in size by 10 % or more (growing polyps), 16 participants had target polyps that had decreased in size by 10 % or more (regressing polyps), and the remaining 28 participants had polyps that were diagnosed as having no change in size. **a** There was a significant inverse correlation between polyp size and the numbers of S100A8+ cells in the polyps. **b** There was a non-significant increase in the density of S100A8+ cells in regressing polyps compared to growing polyps

**Fig. 7:**
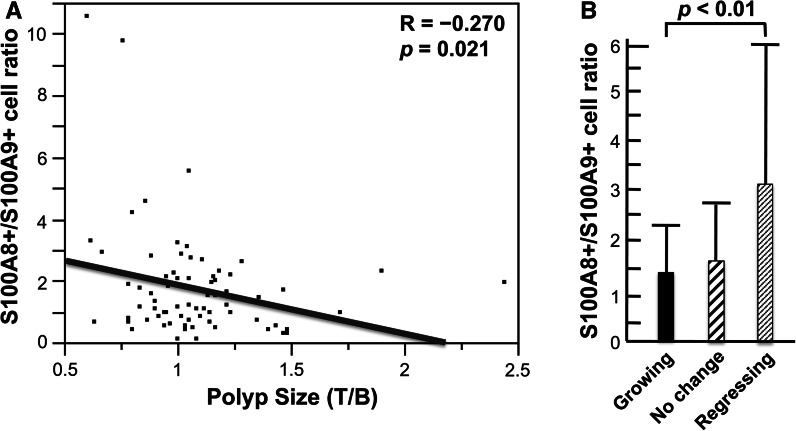
Polyp-associated S100A8+ and S100A9+ cells and polyp growth. At the end of the trial, a final colonoscopic examination was performed. All target polyps were then removed and processed for histological examination. 91 polyps were histologically diagnosed. The density of S100A8+ cells and S100A9+ cells could be determined by immunohistochemistry in 72 of these polyps: 29 participants had target polyps that had increased in size by 10 % or more (growing polyps), 16 participants had target polyps that had decreased in size by 10 % or more (regressing polyps), and the remaining 27 participants had polyps that were diagnosed as having no change in size. **a** There was a significant inverse correlation between polyp size and the ratio of S100A8+/S100A9+ cells in the polyps. **b** The ratio of S100A8+/S100A9+ cells was significantly higher in regressing polyps compared to growing polyps

Figure 5 shows typical S100A8+ cells (panel A) and S100A9+ cells (panel B). There was a significant inverse correlation between polyp size and the density of S100A8+ cells in the polyp (Fig. 6a), and there was an increase, although not significant, of S100A8+ cells in regressing polyps compared to growing polyps (Fig. 6b). There was no correlation between polyp-associated S100A9+ cells and polyp size or growth (data not shown). However, there was a significant inverse correlation between polyp size and the ratio of S100A8+/S100A9+ cells in the polyp (Fig. 7a), and the ratio of S100A8+/S100A9+ cells was significantly higher in regressing polyps than growing polyps (Fig. 7b). S100A8 and S100A9 heterodimerize to form calprotectin and are constitutively expressed at very high levels in neutrophils, in which calprotectin comprises over 40 % of the cytosolic proteins (Goyette and Geczy 2011). S100A8 and S100A9 are also constitutively expressed in myeloid-derived dendritic cells, osteoclasts, hypertrophic chondrocytes, and immature monocytes (Goyette and Geczy 2011). In addition, expression of either one or both of these proteins can be induced in microvascular endothelial cells, keratinocytes, and resident tissue macrophages (Goyette and Geczy 2011). The distribution of S100A8+ and S100A9+ in colorectal polyps is consistent with decreased infiltration of PMNs into regressing polyps and an accompanying increase of S100A8+ cells in these polyps. Human S100A8 and S100A9 are implicated in inflammation, however, the functions of human S100A8 and S100A9 are yet to be precisely defined (Goyette and Geczy 2011; Donato et al. 2013; Lim et al. 2009). In vitro studies with human S100A9 and in vivo studies with animal models indicate that S100A9 can have pro-inflammatory activity: Human S100A9 enhances neutrophil infiltration and retention in extra-vascular tissues (Schnekenburger et al. 2008; Anceriz et al. 2007; Newton and Hogg 1998) and promotes neutrophil degranulation (Simard et al. 2010) and phagocytosis (Simard et al. 2011). Murine S100A8 is also pro-inflammatory (Ryckman et al. 2003; Vandal et al. 2003). In contrast, human S100A8 is reported to have anti-inflammatory activity (Zhao et al. 2011; Lim et al. 2008; Hsu et al. 2005), mediated in part by inhibition of neutrophil infiltration and retention in extra-vascular tissues (Roth et al. 2003; Newton and Hogg 1998; Sroussi et al. 2006). The observation that the ratio of S100A8+/S100A9+ cells correlates with regressing polyps coupled with promotion of neutrophil infiltration by S100A9 and inhibition of neutrophil infiltration by S100A8 is consistent with the observed decrease of PMN infiltration in regressing polyps. The association of inflammation with carcinogenesis (Ichikawa et al. 2011; Gebhardt et al. 2006; Balkwill and Mantovani 2001) coupled with the pro-inflammatory activity of S100A9 and the anti-inflammatory activity of S100A8 is consistent with lower inflammatory potential and lower carcinogenic potential in regressing polyps.

#### Summary

Overall, our observations are consistent with the following paradigm. The colorectal polyps in patients with higher immune system activity had less growth potential, as manifested by their limited growth or regression during the one-year trial period, than polyps in patients with lower immune system activity. One likely mechanism by which the immune system acted was by eliminating polyp cells as they became transformed from pre-cancerous cells into cells with increased tumorigenic potential. In addition, the colorectal polyps in patients with lower inflammatory potential in their colon mucosa had less growth potential than the polyps in patients with greater inflammatory potential in their colon mucosa.

### Effect of bLF on immune parameters

#### Systemic NK cell activity and blood PMN counts and serum hLF

**Fig. 8:**
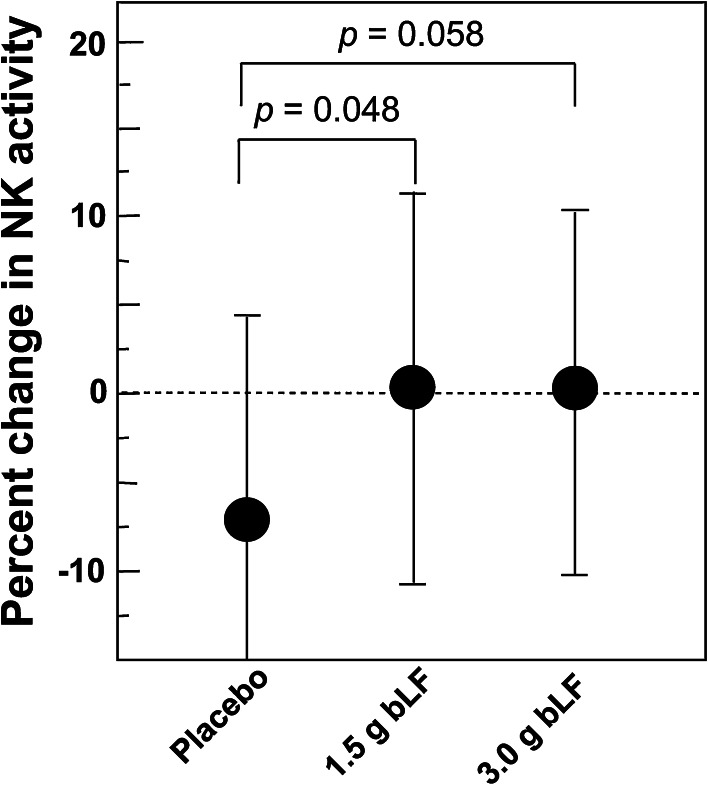
Effect of bLF on systemic NK cell activity. The change in NK activity in peripheral blood samples measured prior to the beginning and at the end of the trial is shown. There were 33 patients in the placebo group, 37 patients in the 1.5 g bLF group, and 32 patients in the 3.0 g bLF group. The relative change in NK activity in the blood of the trial participants at the end of the one-year trial period was significantly higher in the 1.5 g bLF group compared the placebo group; however, the relative change in the 3.0 g bLF group was not significantly increased compared to the placebo group. This figure is adapted from Fig. 2, Kozu et al. (2009), Effect of Orally Administered Bovine Lactoferrin on the Growth of Adenomatous Colorectal Polyps in a Randomized, Placebo-Controlled Clinical Trial

**Fig. 9:**
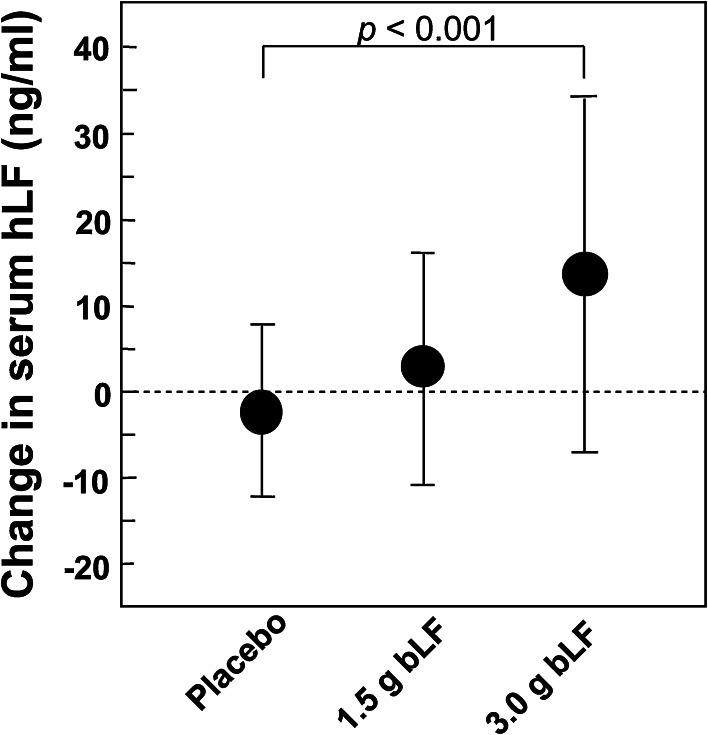
Effect of bLF on serum hLF levels. The change in hLF levels in serum samples measured prior to the beginning of the trial and at the end of the trial are shown. There were 33 patients in the placebo group, 37 patients in the 1.5 g bLF group, and 32 patients in the 3.0 g bLF group. The levels of serum hLF at the end of the one-year trial period was significantly higher in the 3.0 g bLF group compared the placebo group. This figure is adapted from Fig. 1, Kozu et al. (2009), Effect of Orally Administered Bovine Lactoferrin on the Growth of Adenomatous Colorectal Polyps in a Randomized, Placebo-Controlled Clinical Trial

There was an increase in NK activity in the serum of patients ingesting bLF, however, while the increase was statistically significant in participants ingesting 1.5 b bLF, the increase was not significant in participants ingesting 3.0 g bLF (Fig. 8). These incongruent results are consistent with a possible marginal increase in NK activity in participants ingesting bLF. There was also a statistically significant increase in the levels of serum hLF in trial participants ingesting 3.0 g bLF (Fig. 9). (bLF was not detected in the serum of any of the trial participants at any time.) In contrast to serum hLF levels, the increase in the number of PMNs in the blood of participants 63 years old and younger ingesting 3.0 g bLF was not significant (data not shown). As discussed above, since serum hLF is derived primarily from neutrophils (Ambruso et al. 1984; Gessler et al. 2004; Lash et al. 1983; van der Strate et al. 1999; Brown et al. 1986, 1983), the increase in serum hLF levels suggest that in these participants, serum neutrophils had elevated activity. Importantly, blood collection can provide an activating signal for primed neutrophils (Freitas et al. 2008) and as large amounts of lactoferrin are present in neutrophil granules, the presence of primed neutrophils in a blood sample will result in a measurable increase of lactoferrin in the serum obtained from that sample. The possible marginal increase in unstimulated NK cell activity and the significant increase in the activity of neutrophils stimulated by serum collection procedures are consistent with the premise that ingestion of bLF promotes immune function by priming immune effector cells.

**Fig. 10:**
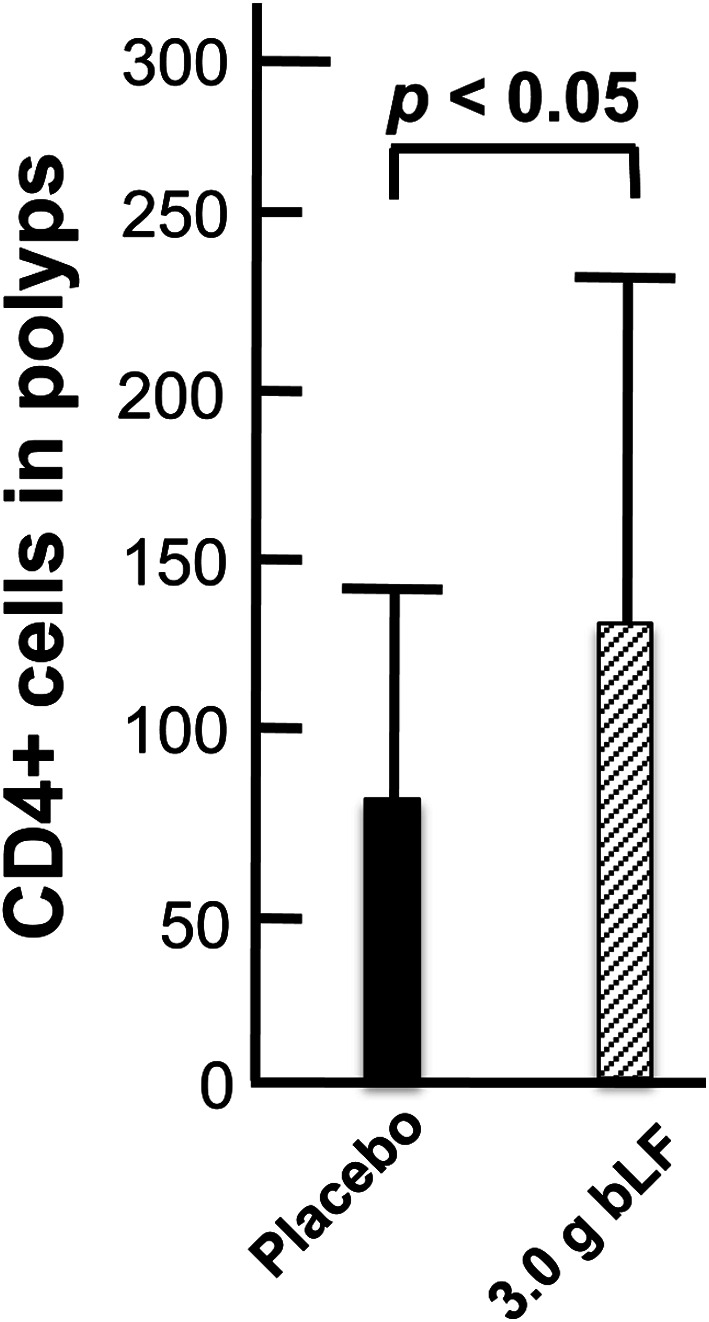
Effect of bLF on polyp-associated CD4+ cells. At the end of the trial, a final colonoscopic examination was performed. All target polyps were then removed and processed for histological examination. 91 polyps were histologically diagnosed, and the density of CD4+ cells was determined in 37 polyps from the placebo group and 37 polyps from the 3.0 g bLF group. There was a significant increase in CD4+ cells in the polyps from the 3.0 g bLF group compared to the placebo group

#### Polyp-associated CD4+ cells

There was a significant increase in CD4+ cells in the polyps of trial participants ingesting 3.0 g bLF (Fig. 10). A likely cause of the increased number of CD4+ in these polyps is induction of cytokine expression in the colons of trial participants ingesting 3.0 g bLF: Using the RNA extracted from the normal mucosal samples collected prior to the beginning and at the end of the trial, we found that ingestion of 3.0 g bLF resulted in an increase of IFNA expression in the colon mucosa (Alexander et al. submitted). These findings are consistent with the increase of peritumoral and intratumoral CD4+ cells 15 days after intralesional injection of basal cell carcinomas with IFNA (Mozzanica et al. 1990) and suggest that induction of IFNA in the colons of participants ingesting 3.0 g bLF resulted in an increase of CD4+ cells in the colon mucosa and, consequently, in the colorectal polyps of these trial participants.

**Fig. 11:**
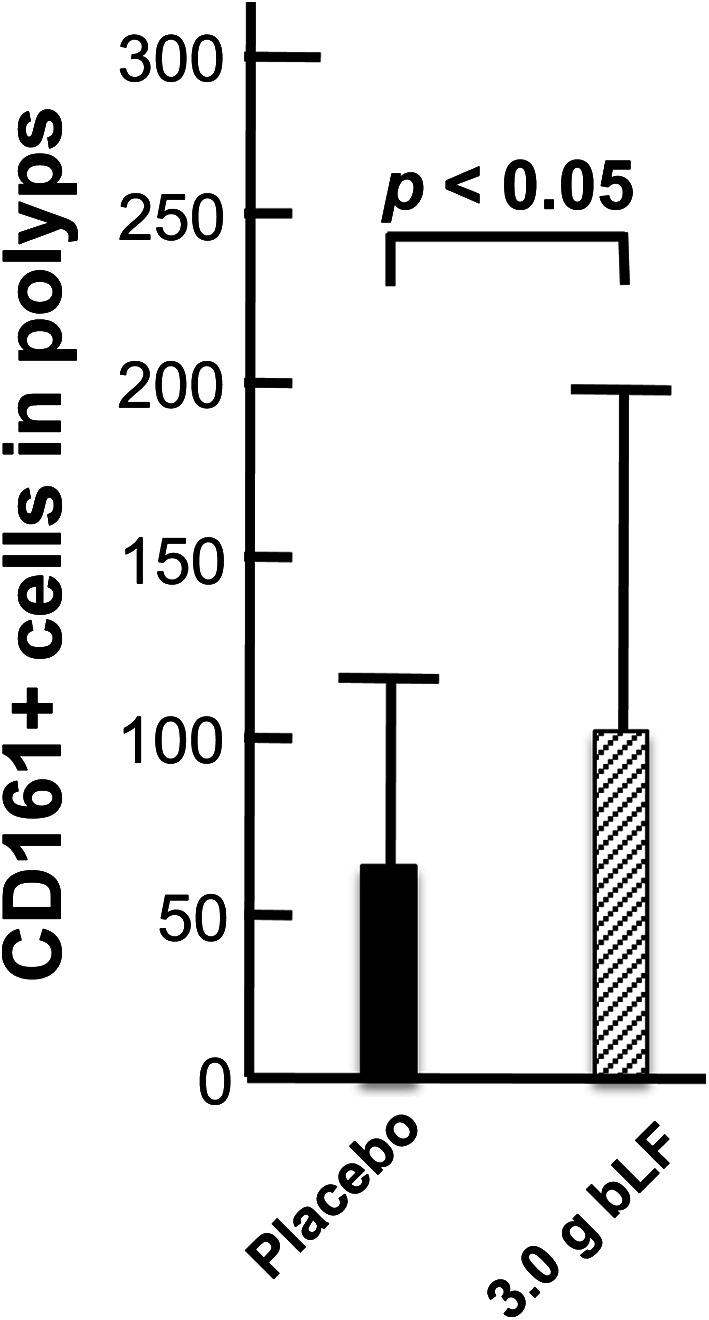
Effect of bLF on polyp-associated CD161+ cells. At the end of the trial, a final colonoscopic examination was performed. All target polyps were then removed and processed for histological examination. 91 polyps were histologically diagnosed, and the density of CD161+ cells was determined in 37 polyps from the placebo group and 35 polyps from the 3.0 g bLF group. There was a significant increase in CD161+ cells in the polyps from the 3.0 g bLF group compared to the placebo group

#### Polyp-associated CD161+ cells

There was also a significant increase of CD161+ cells (primarily NK cells) in the polyps of trial participants ingesting 3.0 g bLF (Fig. 11). It is likely that the increased number of NK cells in these polyps is also caused by bLF-mediated induction of cytokine expression in the colons of these trial participants: IFNA induces expression of CXCL10 (Lande et al. 2003), and CXCL10 is chemotactic for NK cells (Lande et al. 2003; Megjugorac et al. 2004). It is important to note that increased infiltration of NK cells into the polyps of participants ingesting 3.0 g bLF is not equivalent to an increased presence of NK cells in regressing polyps. As discussed above, in the absence of other factors, immune cells involved in immunosurveillance and removal of transformed cells, such as NK cells, will be present in polyps only briefly, as tumorigenic cells are being targeted. However, increased numbers of NK cells in a polyp will enhance the ability of the immune system to target and remove transformed cells in that polyp.

#### Polyp associated PMNs, CD66b+, S100A8+ and S100A9+ cells

There was no significant net effect on the presence of PMNs, CD66+ cells, S100A8+ cells or S100A9+ cells in the polyps of trial participants ingesting 3.0 g bLF (data not shown). This result suggests that ingestion of bLF had little effect on the inflammatory potential of the colon mucosa.

## Summary and conclusions

The data presented in this report show a good correlation between increased levels of NK cell activity in the blood, increased levels of serum hLF, which reflects systemic neutrophil responsiveness, and regression of colorectal polyps. These data are consistent with a correlation between higher immune system activity and suppression of colorectal polyps. Our data also show a good correlation between the presence of CD4+ cells in colorectal polyps and regressing polyps. This finding is consistent with the key role CD4+ cells have in immune system function and is also consistent with a correlation between higher immune system activity and suppression of colorectal polyps. In addition, our data support the proposal that lower inflammatory potential in the colon mucosa, as manifested by decreased numbers of neutrophils and increased numbers of S100A8+ cells, is associated with suppression of colorectal polyps.

Ingestion of bLF caused a possible marginal increase in systemic NK cell activity and a significant increase in serum hLF levels. These findings are consistent with the presence of primed NK cells and neutrophils in the serum. In addition, ingestion of bLF resulted in an increase in the number of CD4+ and NK cells in colorectal polyps. Notably, the colorectal polyps examined in the Tokyo trial persisted throughout the one-year trial period in the patients ingesting 3 g bLF daily, indicating that the polyp-associated CD4+ and NK did not induce an immune response against the polyps. This also consistent with the suggestion that ingestion of bLF primed rather than activated immune effector cells. This premise would predict that ingestion of bLF would not change immune function from OFF to ON, but rather immune function would change from a less responsive state to a more responsive state. Thus, the presence of CD4+ cells and NK cells in polyps without transformed cells would enhance targeting of cells once they acquire a transformed phenotype, thereby decreasing polyp growth. Taken together, our findings are consistent with the proposition that ingestion of bLF suppressed colorectal polyps by enhancing immune system responsiveness.

As previously reported by Kozu et al. (2009), trial participants 63 years old and younger ingesting 3.0 g bLF daily for one year had a statistically significant regression in the growth of colorectal polyps compared with participants ingesting placebo, however, bLF had no significant effect on polyps in participants 64 years old and older. This suggests that while ingestion of bLF resulted in suppression of colorectal polyps, an age related factor affected the trial participants’ response to bLF. One possibility is that to generate an effect, bLF must be digested into peptide fragments, and decreased digestive capability in some of the older trial participants resulted in decreased suppression of colorectal polyps in these individuals. Another possibility, is that immune responsiveness was influenced by the inflammatory potential of the colon mucosa, and the inflammatory potential of the colon mucosa of some of the older trial participants was strong enough to counter the effects of bLF.

As noted above, the colorectal polyps examined in the Tokyo trial persisted throughout the one-year trial period in the patients ingesting 3 g bLF daily. A crucial conclusion that can be drawn from this fact is that bLF did not induce an immune response against the polyps. This is a physiologically reasonable and key consideration: The cells in these polyps were mostly pre-cancerous and, consequently, were essentially normal. Lack of an immune response against these cells indicates that the increased responsiveness of immune effector cells in the trial participants ingesting 3.0 g bLF did not result in an autoimmune response. Importantly, in all of the animal and human studies conducted to date, which include chronic administration to animals and daily intake for one year by human patients, ingestion of bLF has been shown to be toxicologically safe.
